# Conventional machine learning and deep learning in Alzheimer's disease diagnosis using neuroimaging: A review

**DOI:** 10.3389/fncom.2023.1038636

**Published:** 2023-02-06

**Authors:** Zhen Zhao, Joon Huang Chuah, Khin Wee Lai, Chee-Onn Chow, Munkhjargal Gochoo, Samiappan Dhanalakshmi, Na Wang, Wei Bao, Xiang Wu

**Affiliations:** ^1^Department of Electrical Engineering, Faculty of Engineering, Universiti Malaya, Kuala Lumpur, Malaysia; ^2^Department of Biomedical Engineering, Faculty of Engineering, Universiti Malaya, Kuala Lumpur, Malaysia; ^3^Department of Computer Science and Software Engineering, United Arab Emirates University, Al Ain, United Arab Emirates; ^4^Department of Electronics and Communication Engineering, SRM Institute of Science and Technology, Chennai, India; ^5^School of Automation, Guangdong Polytechnic Normal University, Guangzhou, China; ^6^China Electronics Standardization Institute, Beijing, China; ^7^School of Medical Information Engineering, Xuzhou Medical University, Xuzhou, China

**Keywords:** Alzheimer's disease, machine learning, deep learning, convolutional neural network, transformer, classification, neuroimaging, Magnetic Resonance Imaging

## Abstract

Alzheimer's disease (AD) is a neurodegenerative disorder that causes memory degradation and cognitive function impairment in elderly people. The irreversible and devastating cognitive decline brings large burdens on patients and society. So far, there is no effective treatment that can cure AD, but the process of early-stage AD can slow down. Early and accurate detection is critical for treatment. In recent years, deep-learning-based approaches have achieved great success in Alzheimer's disease diagnosis. The main objective of this paper is to review some popular conventional machine learning methods used for the classification and prediction of AD using Magnetic Resonance Imaging (MRI). The methods reviewed in this paper include support vector machine (SVM), random forest (RF), convolutional neural network (CNN), autoencoder, deep learning, and transformer. This paper also reviews pervasively used feature extractors and different types of input forms of convolutional neural network. At last, this review discusses challenges such as class imbalance and data leakage. It also discusses the trade-offs and suggestions about pre-processing techniques, deep learning, conventional machine learning methods, new techniques, and input type selection.

## 1. Introduction

Alzheimer's disease (AD) is a neurodegenerative disease with insidious onset and progressive development. Clinically, AD is characterized by memory disorder, aphasia, apraxia, agnosia, visual skill damage, and general dementia with personality and behavior changes. However, the cause of the disease remains unknown. Currently, there is no accurate diagnosis and validated disease-modifying treatment. In addition, since AD symptoms are sudden and severe memory loss, there is a high cost of caring for the patients. The high increase in public health needs enormous numbers of budget. The socio-economic prices of AD are far more significant than expected. As a result, AD brings a massive burden on the patient's family and society. According to a recent report by Nichols et al. (2022), globally, the number of patients with dementia is 57.4 million in 2019, and the number may increase to around 152.8 million in 2050. So the accurate diagnosis of AD is critical for the patients and society. In general, AD has three stages: normal control (NC), mild cognitive impairement (MCI), and Alzheimer's disease (AD). In particular, MCI is the early stage of AD, which is defined as the intermedia state between AD and normal control. The mark of MCI is loss of memory and poor memory. While some MCI patients proceed to AD, some remain MCI. Early diagnosis is crucial in effective clinical intervention and alleviating disease progression (Livingston et al., 2020). AD/MCI diagnosis is one of the most significant and challenging tasks in AD assessment. The accurate classification of AD/MCI determines the follow-up treatment. What's more, proper treatment during MCI can reduce or slow down the development to AD. So, prediction of conversion from MCI to AD is even more valuable than classification between NC and AD or MCI patients. However, the traditional AD diagnosis methods considerably rely on clinical experts' experience and human efforts. As the development of computer-aided diagnosis, computer softwares can provide automatic classification and prediction of AD. For the reasons mentioned above, computer-aided diagnosis of AD is necessary and significant.

Artificial intelligence has been thriving in recent years, and researchers and engineers conducted extensive research on AD-related areas. According to the methods utilized, these researches are in two categories: convention machine learning and deep learning. Convention machine learning methods contain support vector machine (SVM), random forest, linear regression, naïve Bayesian, artificial neural networks, etc. Deep learning methods include convolutional neural networks, recursive neural networks, etc.

Many biomarkers, such as genetic, biological, and neuroimaging techniques, including Magnetic Resonance Imaging (MRI), fluorodeoxyglucose positron emission tomography (FDG-PET) imaging, amyloid PET, and diffusion tensor imaging (DTI), are used for AD diagnosis. The MRI image is one of the most widely used for the early detection and classification of AD. Since MRI provides high-resolution images of brain anatomical structures, researchers can retrieve rich information from MRI images. MRI shows the shrinkage of brain tissue, particularly the hippocampus, which confirms the structural change in the brain. Moreover, MRI can be used to predict if a patient with MCI will eventually develop Alzheimer's disease since MRI can detect brain abnormalities associated with MCI. In recent years, public open-access databases supplied MRI images of AD biomarkers, and the datasets were maintained by updating and adding new data. Considerable researchers have conducted their work to analyze AD employing MRI-based biomarkers. In this article, we mainly focus on MRI-based applications. Some researchers used MRI together with PET, so we also introduced PET. MRI and PET data as the 3D image which reveals structural brain atrophy are two of the most frequently used modalities in deep learning areas. MRI uses magnetic resonance phenomena to extract electromagnetic signals from the human body and reconstruct a 3D representation of human information. MRI can be done without injecting radioactive isotopes, which makes MRI safer. PET uses short-lived radionuclides to generate images of the target. The PET scanner can detect areas of high radionuclides concentration within the body. Both MRI and PET are non-invasive neuroimaging modalities. The other two most widely used medical tests that evaluate AD levels are the Mini-Mental State Examination (MMSE) and the Clinical Dementia Rating (CDR). Taking the results of MMSE and CDR as the ground truth labels may be incorrect. Still, the results of MMSE and CDR remain valuable references due to the limited biomarkers available.

Multi-modality studies utilize more than one modality of each subject, while single-modality studies use only one modality. Using multi-modality is that features extracted from different modalities could contain complementary information. MRI, FDG-PET, Cerebrospinal Fluid (CSF), MMSE, and Alzheimer's Disease Assessment Scale-Cognitive Subscale (ADAS-Cog) are often-used modalities.

Detecting AD remains a challenging task in computer vision areas for a few reasons. The image dataset is not large enough compared with other image classification datasets like ImageNet. The medical images acquired are usually of low quality, with relatively coarse noise segmentation results. Compared with images in other areas, the complexity of medical images is high. Images are acquired from different devices with various strengths, leading to more effort spent in pre-processing. The distinction between NC and MCI, MCI, and AD are not apparent in computer vision.

The public open-access databases have extensively helped AD-related research in artificial intelligence (A.I.). In recent years, this field has attracted the attention of a considerable number of researchers, and the number of related papers published each year is also boosting rapidly. Therefore, there is a need to analyze and summarize related documents so that researchers can more easily understand the development status of associated fields. We aim to help relevant researchers quickly understand the research status and future trends in related fields. The objectives of this study are to explore the associated datasets, pre-processing techniques, popular conventional machine learning methods, including SVM and RF, and Deep learning methods, including CNN, autoencoders, transformer, and transfer learning. So we examined recent works, compared the trade-off, summarized the current trend, and provided a future guide on computer-aided AD diagnosis using MRI images in the A.I. area. This review mainly focuses on the highly cited studies that adopted the most widely used techniques.

**Figure 1:**
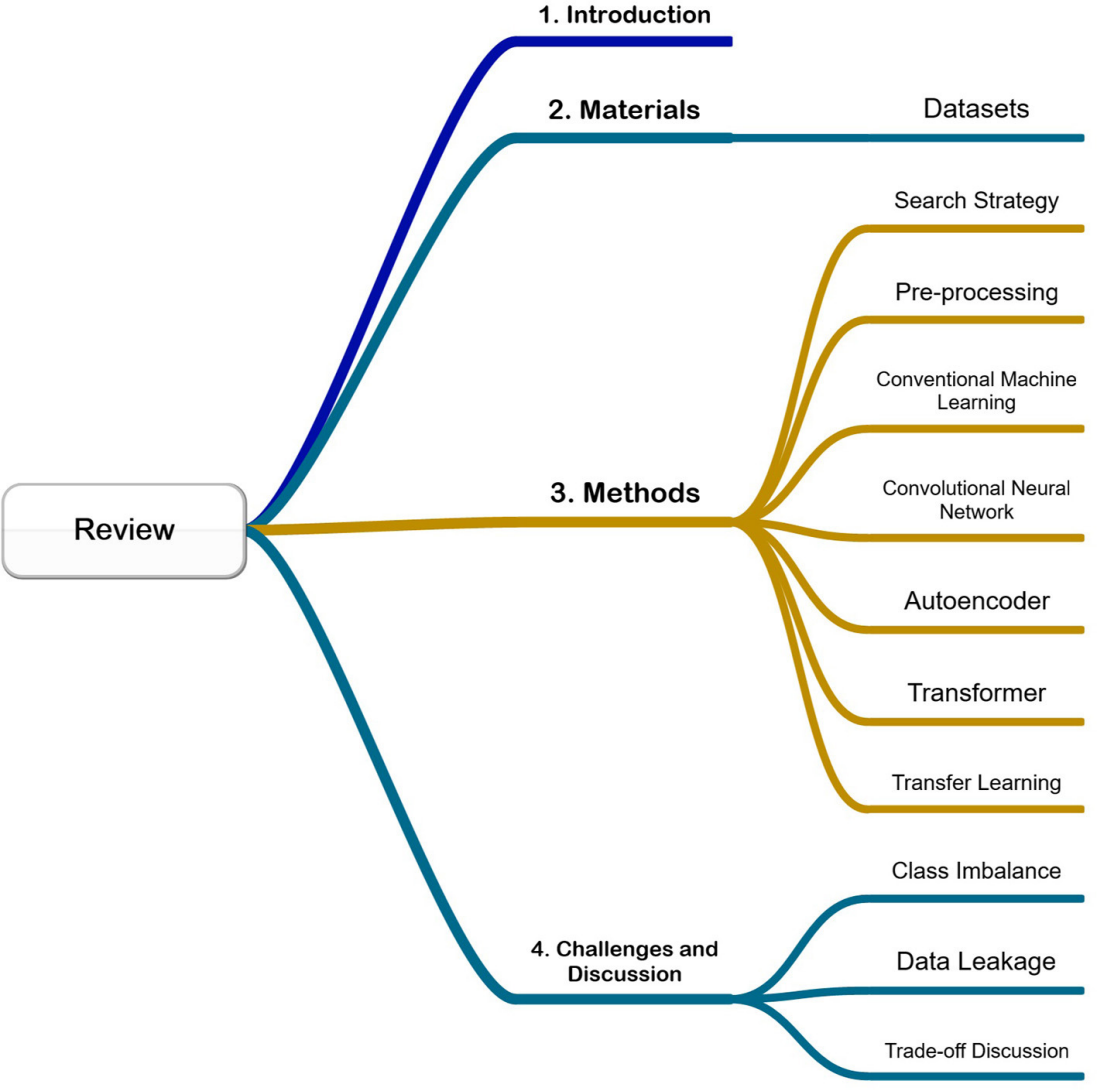
Mind map of this paper.

As shown in Figure 1, we will organize our paper as below: In the Introduction Section, we will have a brief introduction to the background knowledge. In the Materials Section, we will introduce the public datasets that are often used in related areas. In the Methods Section, we will explore our search strategy, the pre-processing techniques, conventional machine learning like Support-vector machine (SVM) and Random forest (RF), convolutional neural network (CNN), autoencoders, transformer, and transfer learning methods. In the Challenges and discussion Section, we will discuss the current challenges like class imbalance, data leakage, and trade-offs when designing a proper model with our recommendations.

## 2. Materials

### 2.1. Datasets

In recent years, many research centers accumulated plentiful medical and image data and published the data to the public. Public data plays a significant role for researchers in research and developing AI on AD. The online datasets make biomarker information like neuroimaging modalities, genetic and blood information, and clinical and cognitive assessments. Most pervasively used datasets include Alzheimer's Disease Neuroimaging Initiative (ADNI) (Jack et al., 2008), Australian Imaging, Biomarker, & Lifestyle Flagship Study of Aging (AIBL) (Ellis et al., 2009), Open Access Series of Imaging Studies (OASIS) (Marcus et al., 2007, 2010; LaMontagne et al., 2019), and Minimal Interval Resonance Imaging in Alzheimer's Disease (MIRIAD) (Malone et al., 2013).

ADNI is notable for being a longitudinal and multicenter study. It is the most common dataset. The objective of ADNI is to investigate if the combination of MRI, PET, other biological markers, and clinical and neuropsychological assessment could measure the progression of MCI and early AD. ADNI-1, ADNI-GO, ADNI-2, and ADNI-3. The following collections are the supplement and improvements of previous ones. From patients, ADNI researchers collect several data types, including clinical, genetic, MRI, PET images, and biospecimen. ADNI-1 contains 200 NC, 400 MCI, and 200 AD. ADNI-GO adds 200 MCI on ADNI-1. ADNI-2 extends ADNI-1 and ADNI-GO with 150 NC, 100 early MCI, 150 late MCI, and 150 AD. ADNI-3 expands existing ADNI-1, ADNI-GO, and ADNI-2, adding 133 NC, 151 MCI, and 87 AD.

AIBL collects imaging and medical data from 211 individuals with AD, 133 individuals with MCI, and 768 healthy individuals without cognitive impairment.

OASIS aims to share neuroimaging brain data sets with researchers in related areas. OASIS has three releases: OASIS-1 contains 434 MRI scans from 416 subjects. OASIS-2 contains 373 MRI scans from 150 subjects. OASIS-3 contains 2,168 MRIs and 1,608 PET scans from 1,098 subjects.

The MIRIAD dataset contains 708 MRI scans from 46 AD patients and 23 NC volunteers.

Moreover, some studies use the datasets above along with their own datasets. For instance, Basaia et al. (2019) collected 3D T1-weighted images from 124 patients with probable AD, 50 patients with MCI, and 55 healthy controls. They named their dataset as “Milan” dataset. Suk et al. (2016b) used images from ADNI-2 and their in-house dataset with 37 participants of 12 MCI subjects and 25 NC subjects.

## 3. Methods

This section reviews a few classical conventional machine learning and deep learning methods. Firstly, we introduce the search strategy for our review. Secondly, we will examine two traditional machine learning methods: Support-vector machine and random forest. Thirdly, we will review the convolutional neural network, including popular CNN backbones and different input types of CNN. Fourthly, we will discuss autoencoders in AD detection. Fifthly, we talk about transformer. At last, we will briefly introduce the application of transfer learning.

### 3.1. Search strategy

This paper is conducted by following the PRISMA 2020 guidelines (Page et al., 2021).

#### 3.1.1. Databases and keywords of search

We searched Scopus, one of the largest abstract and citation databases of peer-reviewed literature: scientific journals, books, and conference proceedings. We selected research papers regarding Alzheimer's disease diagnosis using AI techniques from 2013 to 2022. Scopus searches within the article title, abstract, and keywords. The papers will not be selected if the search documents only appear in the text or figure caption.

##### 3.1.1.1. Inclusion keyword groups

Inclusion Keyword group 1: “Alzheimer's disease” OR “AD” OR “dementia” OR “mild cognitive impairment” OR “MCI.”

Inclusion Keyword group 2: “Artificial intelligence” OR “AI” OR “machine learning” OR “deep learning” OR “computer-assisted diagnosis” OR “computer assisted diagnosis” OR “CAD” OR “Neural network” OR “convolutional neural network” OR “CNN” OR “recurrent neural network” OR “RNN” OR “random forest” OR “support vector machine” OR “SVM.”

Inclusion Keyword group 3: “Magnetic Resonance Imaging” OR “MRI” OR “Structural Magnetic Resonance Imaging” OR “sMRI” OR “Functional Magnetic Resonance Imaging” OR “fMRI.”

##### 3.1.1.2. Exclusion keyword groups

Exclusion Keyword group 1: “Schizophrenia” OR “depression” OR “major depressive disorder.”

Exclusion Keyword group 2: “Computed tomography” OR “CT” OR “Positron Emission Tomography” OR “PET” OR “amyloid-β.”

Exclusion Keyword group 3: “REVIEW.”

Initially, the search result contained 2,561 documents in total. Then we filtered the document type as article (1,712 documents) and source type as “journal” (1,705 documents). At last, we gave up those documents written in languages other than English (1,678 documents).

##### 3.1.1.3. Exclusion criteria

1. The initial search result is further filtered according to the following exclusion criteria.
2. Studies only focus on preprocessing, brain extraction, or other similar feature selection.
3. Studies using other biomarkers only other than MRI images (e.g., CT, PET, amyloid-β, genetic, etc.)
4. Studies focus on brain aging or other types of brain disease.
5. Conference Paper, conference review, book chapter, editorial, review, note, letter, or data paper.
6. Conference proceeding, book series, book, or trade journal.
7. Articles are written in languages other than English.

**Figure 2:**
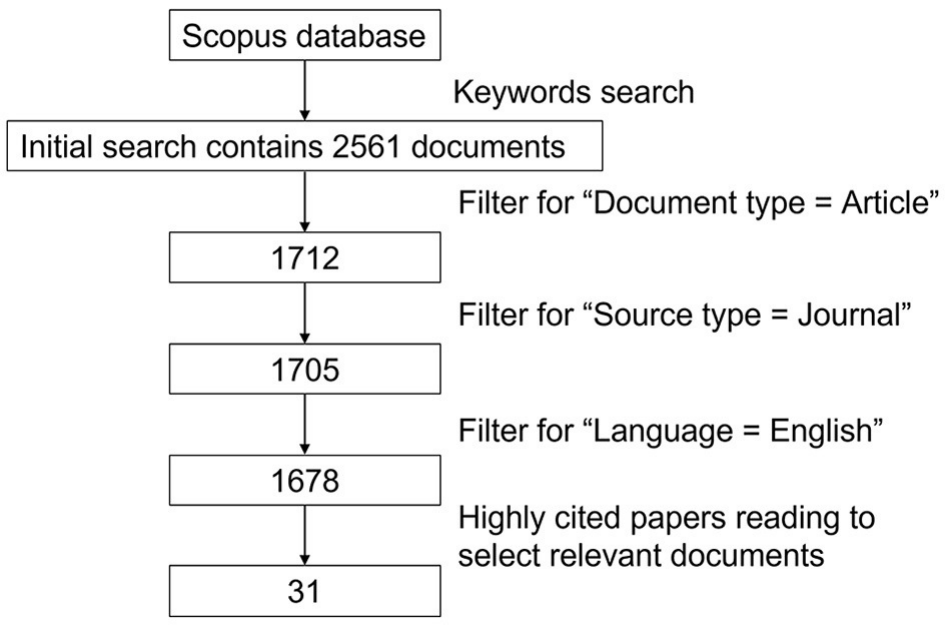
Paper search flowchart.

The criteria above generated a collection of 31 articles in total for in-depth reviewing as shown in Figure 2.

### 3.2. Pre-processing

The size of the training set highly impacts classification performance. In all datasets introduced above, the numbers of image scans retrieved from AD and MCI subjects are limited. In most studies, pre-processing must be done before manipulating the data. Pre-processing is a set of image processing tasks performed on the acquired image scans. Some MRI software packages like FreeSurfer (Fischl, 2012), Computational Anatomy Toolbox (CAT12), FMRIB Software Library (FSL) (Jenkinson et al., 2012), Statistical Parametric Mapping (SPM), ANTS (Avants et al., 2009), etc., provide well-encapsulated pre-processing algorithms. Pervasively used pre-processing techniques include registration, normalization, smoothing, segmentation, skull-stripping, noise removal, temporal filtering, covariates removal, etc. This review will introduce intensity normalization, registration, skull-stripping, tissue segmentation, and class balancing.

#### 3.2.1. Intensity normalization

Intensity normalization, known as field correction or intensity inhomogeneity correction, refers to rescaling the intensities of each pixel to a normalized intensity. In the process of MR image acquisition, various scanners or parameters will scan distinct subjects or the same subject at different times, which may cause significant intensity changes. Large intensity changes will significantly affect the performance of subsequent pre-processing like registration and segmentation.

#### 3.2.2. Registration

Registration is a method to spatially align image scans to ensure the correspondence of anatomy across modalities, individuals, and studies. Registration is also used in multi-modality tasks for co-registration. The most commonly used templates are MIN 305, Collin27, and MNI152. Liu et al. (2016) reported a higher performance adopting multiple templates over a single template. They utilized multiple templates for feature extraction, selected the most representative features of each template, trained multiple SVM classifiers, and ensemble the results of all classifiers to generate the result. However, multiple templates lead to high computational costs, especially in image registration.

#### 3.2.3. Skull-stripping

Skull-stripping or brain extraction means removing the non-brain tissues like skull, fat, eyes, etc., and remaining gray matter (GM), white matter (WM), Cerebrospinal fluid (CSF), etc. in the brain scan.

#### 3.2.4. Tissue segmentation

Tissue segmentation means partitioning the image scan into segments corresponding to various tissues. The volume of tissues is a measurement often used after tissue segmentation. GM probability maps are a popular input form in classification tasks. Usually, Pre-processing techniques like intensity normalization and registration need to be done.

#### 3.2.5. Data augmentation

Data augmentation is a way to solve the limitation on the number of subjects in a dataset. It is a technique to enlarge the dataset without collecting new data by generating new data samples from the existing data. Data augmentation techniques have been used, including cropping, reflection, random translation, gamma correction, scaling, random rotation, elastic transform, vertical flip, horizontal flip, and different types of blurring. Moreover, new synthesis techniques like autoencoders and generative adversarial networks are also used in data augmentation. However, synthesis techniques need more proof of the effectiveness of the generated images in AD-related classification and prediction tasks.

### 3.3. Conventional machine learning

Support-vector machines (SVMs) are supervised learning methods in conventional machine learning and are often used to solve classification and regression problems. SVMs map the input to points in multidimensional space to maximize the margin between hyperplanes of different data types. A kernel function, for example, Gaussian or polynomial function, maps the current multidimensional space into a higher-dimensional space. SVMs can be used alone and work with other methods for both conventional machine learning and deep learning methods. Since SVMs can achieve a relatively good performance and the principles of SVMs work are clear and understandable, SVMs are extensively applied in industrial and scientific areas. Suk et al. (2016a) used a linear SVM classifier, and Suk and Shen (2013) and Suk et al. (2015) used multi-kernel SVMs to classifier integrated features from multi-modal inputs. Shi et al. (2018) proposed a model that takes stacked deep polynomial networks (DPN) as the feature extractor and a linear kernel SVM as the classifier. Suk et al. (2014) used a linear SVM for the hierarchical classifiers to work with feature representations found by Deep Boltzmann Machine (DBM).

The multi-kernel SVMs provide more flexibility than the single kernel SVM. Although multi-kernel SVM has shown excellent performance in many tasks, efficiency is the most significant bottleneck for developing multi-kernel SVM. The computational complexity and difficulty of multi-kernel SVMs are much more significant than single kernel SVM. In terms of space, the multi-kernel SVM algorithms need to calculate the kernel combination coefficients corresponding to each kernel matrix, so multi-kernel matrices must participate in the operation. In other words, multi-kernel matrices need to be stored in memory simultaneously. If the number of samples is too large, the dimension of the kernel matrix will be huge. If the number of kernels is also too large, it will undoubtedly occupy colossal memory space. In terms of time, training of multi-kernel SVM is time-consuming. The high time and space complexity are one of the main reasons the multi-kernel SVM algorithms cannot be widely used. Suk and Shen (2013) and Suk et al. (2015) used multi-kernel SVM classifiers in the model to deal with the feature vectors extracted from Stacked AEs. Khedher et al. (2015) reported an accuracy of 88.49%, specificity of 91.27%, and sensitivity of 85.11% using partial least squares and PCA as feature extractors and linear and RBF kernel SVM as classifiers.

Random forest (RF) is an ensemble algorithm. Each decision tree is a classifier. Multiple decision tree classifiers form the random forest. Individual decision trees are trained in parallel. Random forest integrates all classification voting results and assigns the category with the most votes as the final output. Random forest is a flexible and practical method. It works well on a large dataset. It can handle thousands of input variables without dimension reduction. It estimates the significance of different variables in a task. Calculating many trees and integrating their outputs can consume many computing resources. Moradi et al. (2015) proposed a novel biomarker-based diagnosis in classifying different stages of MCI by utilizing a low-density separation classifier and a random forest classifier. Lebedev et al. (2014) tested random forest on ADNI and AddNeuroMed datasets using MRI images and a combination of morphometric measurements with ApoE-genotype and demographics (age, sex, and education) MRI images. Bi et al. (2020) aimed to overcome the minor sample issue and proposed a clustering evolutionary random forest architecture to deal with multimodal data from ADNI to detect abnormality in the brain and pathogenic genes.

### 3.4. Convolutional neural network

Deep learning is a subset of machine learning techniques in which the learning process is performed through a hierarchical and deep structure. Deep learning techniques have received significant attention in the last few years and have been used widely in different brain studies. One of the most successful deep learning methods is the convolutional neural network.

Convolutional Neural Networks (CNN) are artificial neural networks that use convolution operations to filter the input data and extract useful features. Research on CNN has emerged and thrived swiftly. CNN has attracted widespread attention from researchers and achieved state-of-the-art results on various tasks in detection, classification, and segmentation problems in different domains, including medical imaging, natural language processing, etc. The tremendous success CNN achieved in the classification and segmentation of realistic images has promoted the development and application of CNN in the medical area. In recent years, CNN has performed well in organ segmentation and disease detection tasks. The classic CNN structure consists of a series of convolutional layers, pooling layers, activation layers, and fully connected layers. A SoftMax function is applied to classify the input image with probabilistic values between zero and one.

The convolutional layer contains concepts of local receptive fields, shared weights, filters, stride, and padding. A filter contains unknown parameters that will be learned during training. The convolution is the process by which a filter slides across the whole image from top-left to bottom-right and convolves with the input image to calculate the weighted sum. The stride refers to the step size that a filter moves in per slice. However, the edges' pixels will never be in the center of a filter, and a filter cannot extend beyond the edge region. After each convolution between the input and the filter, only part of the pixels is detected at the edge, and information at the image boundary is lost. Padding is designed to overcome this issue. Padding means filling in some values along the input boundaries to increase the input size. Usually, the values filled are zeros. Padding is needed when it is necessary to keep the dimensions constant before and after convolution to avoid information loss. The size of the filters determines the receptive field in the convolutional layers. The convolutional layers are excellent feature extractors for images since images contain massive spatial redundancy, and convolutional layers solve this characteristic of images with shared weights. After reducing spatial redundancy, the feature vector that the convolution layers output stands for the image's content.

The pooling layer is the dimension reduction operation on the feature maps. It helps reduce the number of parameters to train and accelerates the training process. The most widely used pooling layers are max pooling, average pooling, and global pooling. Max pooling outputs the maximum value within the region of the feature map covered by the filter. Average pooling calculates the average value of the elements presented within the feature map region covered by the filter. Global pooling reduces each channel in the input to a single value.

The activation layer provides a non-linear mapping to the output of the convolutional layer. The calculations in a convolutional layer are linear. The non-linearity provided by activation layers enhanced the reasoning ability of the network. The most pervasively used activation functions include ReLU, Sigmoid, Tanh, etc.

The fully connected layer takes the feature extractor's inputs and predicts the correct label with probabilities.

CNN can be used as the feature extractor and classifier or only feature extractor. Some researchers use CNN to extract features and adopt the conventional machine learning method for classification. Suk et al. (2017) utilized CNN to take the target-level representations generated from the sparse regression for clinical decision making. Feng et al. (2020) applied 3D CNN with MRI to execute AD classification using MRI images. They replaced SoftMax with an SVM as the classifier, and this 3D-CNN-SVM model achieved better classification performance than 2D-CNN and 3D-CNN. With the thriving of CNN in computer vision, researchers contribute several CNN backbones that achieve state-of-the-art performance in many tasks.

When comparing conventional machine learning and deep learning methods in AD-related areas, we can conclude that: in general, deep learning methods achieve better performance than conventional machine learning methods. The proper size of the training samples should be no < 1,000. A dataset containing over five thousand samples can be considered sufficient to train a deep learning model that achieves high accuracy (Zhao et al., 2021).

#### 3.4.1. CNN backbones

CNN backbones refer to the feature extracting networks or feature extractors. In this section, we will introduce classic CNN backbones that are pervasively used in AD diagnosis tasks.

##### 3.4.1.1. LeNet

LeCun et al. (1998) proposes LeNet, the first work that uses CNN in a character recognition task. The basic concepts of convolutional, pooling, and fully connected layers are introduced in one architecture. It also introduces the idea of local receptive fields within CNN. These concepts are the fundamentals of the other deep learning module. Yang and Liu (2020) propose their model with LeNet-5 to do classification and prediction. They take PET images of 350 subjects who are MCI from ADNI. The model achieves sensitivity and specificity of 91.02 and 77.63% in MCI transformation prediction.

##### 3.4.1.2. AlexNet

A significant architecture after LeNet (Krizhevsky et al., 2017) proposed AlexNet. A Rectified Linear Unit (RELU) was used as the activation function. Besides, the author introduced a way to train the networks using multiple GPUs.

##### 3.4.1.3. VGG

Simonyan and Zisserman (2015) proposed VGG. A stack of 3 × 3 convolution filters was used to replace large convolution filters like 5 × 5, 7 × 7, 9 × 9, or 11 × 11 convolution filters. A stack of small convolution filters for a given receptive is better than one large convolution filter. The use of small filters results in fewer parameters and deeper networks which will help train a more complex model in a shorter time. Jain et al. (2019) utilized the transfer learning approach to build the AD classification model. The feature extractor in this work was VGG16 which was pre-trained on ImageNet. They converted 3D MRI images to 2D slices, selected the most informative 32 slices in pre-processing, and then fed the slices into VGG16, followed by fully connected layers. Although their dataset had MRI images of 150 subjects from ADNI, the model achieved an accuracy of 99.14, 99.30, and 99.22% for AD vs. CN, AD vs. MCI, and MCI vs. CN classifications. Even though the classification accuracy was high for all binary tasks, the generality of the proposed model was highly doubted since the dataset was too small. Lim et al. (2022) tested a CNN, VGG-16, and ResNet-50 as the feature extractor to distinguish NC, AD, and MCI using MRI images. They trained the CNN from scratch and pre-trained VGG-16 and ResNet-50 on the ImageNet database. VGG achieved the best performance with an accuracy of 83.90%, precision of 82.49%, recall of 83.90%, and F1-score of 83.19%.

##### 3.4.1.4. GoogLeNet

Szegedy et al. (2015), Szegedy et al. (2016), and Szegedy et al. (2017) contributed several versions of the Inception structure and introduced a series of new ideas, including the Inception module and batch normalization. Instead of choosing whether we should use 3 × 3, 5 × 5, or 7 × 7 filters manually, the inception structure automatically makes the network learn how to find a proper structure. Batch normalization introduced in inception v2 reduces internal covariate shift, which is generated after convolution operations. The consistency of statistical characteristics of data is maintained during training. Inception v3 further replaces the large convolution kernel with the small convolution kernel. A convolution kernel of n × n is cracked into a stack or parallel form of 1 × n and 1 × n convolution kernel. General network design principles suggested in inception v3 are slowly reducing the information's dimension to the desired extent. Ding et al. (2019) used Inception v3 pre-trained on ImageNet as their deep learning backbone. They collect 2,109 PET images of 1,002 patients from ADNI as their dataset.

##### 3.4.1.5. ResNet

He et al. (2016) proposed the deep residual neural networks (ResNet) to deal with problems of vanishing and exploding gradients. Before ResNet came into being, a network could not be designed as deep since the gradient vanishes quickly as the network goes deeper. The network can extract more complex feature patterns when increasing the number of network layers. Theoretically, when a model becomes deeper, better results should be obtained. However, the network accuracy becomes saturated or even decreases as the network depth increases. ResNet solves this issue by adding shortcut connections that skip one or more layers. The accumulation layer only does the identity mapping when the residual is zero. At least the network performance will not decline. The residual will not be zero, enabling the accumulation layer to learn new features based on the input features. Usually, the residual will be relatively small, so the model is easy to train. Abrol et al. (2020) applied a 3D ResNet in their network for classification and prediction. They took 3D gray matter images as the input to train the model for MCI detection first, then utilized transfer learning to transfer the trained model to the domain of NC and AD classification. Korolev et al. (2017) adopted a 3D ResNet and a CNN network similar to VGG to extract features necessary for 3D image classification using brain MRIs. Both networks worked well to classify AD and NC but failed to separate AD and NC from MCI. Islam and Zhang (2018) tested an architecture that ensembled Inception v4 and ResNet to identify different stages of AD and achieved an accuracy of 93.18% on OASIS.

##### 3.4.1.6. DenseNet

Huang et al. (2017) proposed DenseNet to make full use of features from all layers. Two main approaches to improving neural effects are going deeper and becoming more expansive. On the contrary, DenseNet connects all layers directly. In other words, the input for each layer is derived from the output for all previous layers. By doing so, DenseNet mitigates vanishing gradient and makes the best use of features to improve the effect. At the same time, the number of parameters is reduced to some extent. Wang et al. (2019) proposed their model in which every classifier takes the 3D DenseNet as the backbone, followed by fully connected layers and a softmax function. Each 3D DenseNet is initialized and trained separately. A voting system is adopted to integrate the probabilistic scores generated from independent classifiers. The model is trained on images of 833 subjects in the ADNI dataset. Liu et al. (2020) integrated the multi-task deep CNN and DenseNet models for hippocampal segmentation and AD classification. In detail, the multi-task deep CNN extracted the features for segmentation and classification, and a 3D DenseNet learned the features for disease classification. At last, the model integrated the features learned from the multi-task CNN and DenseNet models to make the classification. Wang S. et al. (2018) ensembled 3D-DenseNets for AD and MCI diagnosis. They adopted DenseNet due to the issue of limited data and trained a few 3D-DenseNets with varying hyperparameters. The final result is generated with the weighted sum of each base 3D-DenseNets, and the model achieved an accuracy of 97.19%. Zhang et al. (2021) also proposed their network using 3D DenseNet. Usually, training a deep learning model like DenseNet with such a small dataset usually results in a high risk of overfitting. The voting strategy help compensates for this fault. However, training multiple deep learning models from scratch is time-consuming and inefficient. Transfer learning may be a good choice.

#### 3.4.2. Input types management

CNN is a powerful tool that can process features in different sizes and dimensions. Based on four different input types, four main categories of methods are pervasively used in CNNs: 2D slice-based, 3D patch-based, 3D region-of-interest-based (ROI-Based), and 3D subject-level. Table 1 presents comparisons among recent works.

**Table 1:** Comparison among papers with high citations in AD diagnosis.

**References**	**Scan type**	**Dataset**	**Subjects**	**Participants**	**Accuracy**	**Technical details**	**Pre-processing**
Suk and Shen (2013)	MRI + PET	ADNI	202	HC: 52, AD: 51, MCI: 99	98.8%	Stacked AEs + a multi-kernel SVM	Anterior commissure-posterior commissure correction, skull-stripping, cerebellum removal, and tissue segmented
Liu et al. (2014)	MRI + PET	ADNI	311	HC: 77, AD: 65, pMCI: 67, sMCI: 102	91.4%	Stacked sparse AEs + a softmax layer	Non-linear registration and tissue segmentation
Lebedev et al. (2014)	MRI	ADNI AddNeuroMed	896	HC: 225, MCI: 165, AD: 185 HC: 100, AD: 107, MCI: 114	Overall Accuracy ADNI: 86.6% AddNeuroMed: 86.25%	RF	FreeSurfer segmentation and cortical reconstruction
Suk et al. (2014)	MRI + PET	ADNI	398	HC: 101, AD: 93, MCI: 204	95.35%	DBM + a linear kernel SVM	Anterior Commissure (AC)-Posterior Commissure (PC) correction, skull-stripping, cerebellum removal, and tissue segmented
Payan and Montana (2015)	MRI	ADNI	2,264	HC: 755, AD: 755, MCI: 755	95.39%	Sparse AEs and 3D CNN + FC	Normalization
Suk et al. (2015)	MRI + PET	ADNI	202	HC: 52, AD: 51, pMCI: 43, sMCI: 56	89.13%	Stacked AEs + a multi-kernel SVM	Anterior commissure (AC)-posterior commissure (PC) correction, skull-stripping, and cerebellum removal.
Moradi et al. (2015)	MRI	ADNI	825	HC: 231, MCI: 394, AD: 200	75%	LDS + RF	Intensity correction, spacial normalization, and tissue segmentation
Khedher et al. (2015)	MRI	ADNI	818	HC: 229, AD: 188, MCI: 401	88.49%	Partial least squares+ PCA + SVM	Spatial Normalization and segmentation(GM, WM, CSF)
Li et al. (2015)	MRI + PET + CSF + MMSE + ADAS-Cog	ADNI	202	HC: 52, AD: 51, MCI: 99	91.4%	PCA features stacked RBMs + a linear kernel SVM	Anterior commissure-posterior commissure correction, skull stripping, cerebellum removal, and spatially normalization
Suk et al. (2016a)	MRI	ADNI-2 In-house dataset	100	HC: 31, MCI: 31 HC: 25, MCI: 13	72.58% 81.08%	Deep Auto-Encoder	Realignment and normalization
Hosseini-Asl et al. (2016a)	MRI	ADNI, CADDementia	310 + 30 = 240	HC: 70, AD: 70, MCI: 70 30 subject	AD vs. MCI vs. NC: 94.6% AD + MCI vs. NC: 95.7% AD vs. NC: 99.3% AD vs. MCI: 100% MCI vs. NC: 94.2%	A 3D CNN pre-trained with stacked 3D convolutional AEs	Normalizing, skull stripping, and intensity normalization
Hosseini-Asl et al. (2016b)	MRI	ADNI, CADDementia	310 + 30 = 240	HC: 70, AD: 70, MCI: 70 30 subject	AD vs. MCI vs. NC: 89.1% AD + MCI vs. NC: 90.3% AD vs. NC: 97.6% AD vs. MCI: 95% MCI vs. NC: 90.8%	A 3D CNN pre-trained with stacked 3D convolutional AEs	Normalizing, skull stripping, and intensity normalization
Liu et al. (2016)	MRI	ADNI	459	HC: 128, AD: 97, sMCI: 117, pMCI: 117	93.06%	Ensemble SVMs	Non-parametric non-uniform bias correction, skull stripping, cerebellum removal, tissue segmentation, and affine alignment
Suk et al. (2017)	MRI	ADNI	805	HC: 226, AD: 186, pMCI: 167, sMCI: 226	AD vs. NC: 90.28% MCI vs. NC: 74.20% pMCI vs. sMCI: 73.28%	Sparse regression + CNN	Anterior Commissure (AC)-Posterior Commissure (PC) correction, skull-stripping, and cerebellum removal
Korolev et al. (2017)	MRI	ADNI	231	HC: 61, AD: 50, sMCI: 77, pMCI: 43	88%	3D CNN based on ResNet and VGGNet +	Alignment and skull stripping
Sarraf et al. (2017)	MRI	ADNI	144 + 302 = 446	HC: 92 + 91, AD: 52 + 211	100%	GoogLeNet and LeNet-5 +	Skull stripping, tissue segmentation, registration, and smoothing
Islam and Zhang (2018)	MRI	OASIS	416	416	93.18%	2 CNNs, Inception v4,ResNet	Data augmentation
Lin et al. (2018)	MRI	ADNI	818	HC: 229, AD: 188, MCI: 401	79.90%	PCA + Lasso + CNN	Skull-stripping, deformation registration, and intensity normalization
Wang S. H. et al. (2018)	MRI	OASIS, local	196	HC: 98, AD: 28 AD: 70	97.65%	A 2D CNN	Brain extraction, spatial normalization, normalization, smoothing, and histogram stretching
Shi et al. (2018)	MRI + PET	ADNI	202	HC: 52, AD: 51, sMCI: 56,breakpMCI: 43	97.13%	Stacked DPN +a linear kernel SVM	Anterior commissure (AC)-posterior commissure (PC) correction, intensity inhomogeneity, skull-stripping, cerebellum removal, tissue segmentation, registration
Basaia et al. (2019)	MRI	ADNI 1,2,GO + Milan dataset	1,385	In total HC: 407, AD: 418, c-MCI: 280, sMCI: 280 ADNI HC: 352, AD: 294, MCI: 763 Milan HC: 55, AD: 124, MCI: 50	AD vs. HC: 99% on ADNI 98% on ADNI + Milan cMCI vs. sMCI: 75% on both datasets	CNN	Spatial Normalization and tissue segmentation,
Wang et al. (2019)	MRI	ADNI	833	HC: 315, AD: 221, MCI: 297	97.52%	Esemble 3D-CNN	Grad-warping, intensity correction, skull stripping, and alignment
Khan et al. (2019)	MRI	ADNI	150	HC: 50, AD: 50, MCI: 50	99.20%	VGG	Employ image entropy to select the most informative slices
Jain et al. (2019)	MRI	ADNI	150	HC: 50, AD: 50, MCI: 50	95.73%	VGG-16 pre-trained on ImageNet + 2D CNN + FC	Motion Correction, non-uniform intensity normalization, Talairach transform computation, intensity normalization, and skull stripping
Liu et al. (2020)	MRI	ADNI	449	HC: 119, AD: 97, MCI: 233	AD vs. NC: 88.9% MCI vs. NC: 76.2%	3D DenseNet	Hippocampus segmentation and affine registration Tissue segmentation and non-linear registration
Lian et al. (2020)	MRI	ADNI-1 ADNI-2	951	HC: 229, AD: 199, sMCI: 226, pMCI: 167 HC: 200, AD: 159, sMCI: 239, pMCI: 38	AD vs. NC: 90.3% pMCI vs. sMCI: 80.9%	FCN	Anterior commissure (AC)-posterior, commissure (PC) correction, intensity correction, skull stripping, cerebellum removing, and affine registration
Abrol et al. (2020)	MRI	ADNI	828	HC: 237 AD: 157 sMCI: 245 pMCI: 189	83.01%	CNN +3D ResNet	Tissue segmentation, normalization, and smoothing
Feng et al. (2020)	MRI	ADNI	489	HC: 179, AD: 153, MCI: 157	NC: 93.71% MCI: 96.82% AD: 96.73%	3D CNN +SVM	Spatial normalization, skull stripping, tissue segmentation, affine transition, and registration
Bi et al. (2020)	MRI	ADNI	72	HC: 35, AD: 37	90%	RF	Normalization and smoothing
Odusami et al. (2021)	MRI	ADNI2	113	HC: 25, AD: 25, MCI: 63	EMCI vs. AD: 99.99% LMCI vs. AD: 99.95% MCI vs. EMCI: 99.95%	ResNet18	Random resize, cropping, random rotation, random horizontal flip, center cropping, and normalization
Liu et al. (2021)	MRI	OASIS ADNI	430	HC: 332, AD: 30, MCI: 68	78.02%	Deep separable CNN	Data enhancement processing (clipping, flipping, increase contrast, rotate etc.)

##### 3.4.2.1. 2D slice

2D slice-based approaches extract 2D slices from a 3D image to reduce the number of hyper-parameters. The hypothesis here is useful features for classification or prediction tasks can be extracted from 2D slices. A common way to extract 2D slices from a 3D image is to project the whole brain scan to the sagittal, coronal, and axial planes. Sometimes the sagittal, coronal, and axial planes are also called the median, frontal, and horizontal planes. The center part of the brain is usually more informative than the parts on the edges. The information entropy of the images in the center part is larger than the rest. As a result, not all slices will be used during training. Slices of sagittal, coronal, and axial views contain complementary information. Some studies integrate features extracted from sagittal, coronal, and axial views. It is easy to obtain large numbers of samples when using 2D slices. A deep learning model with 2D CNN usually contains fewer parameters and needs a shorter training time than a 3D model. The disadvantage of slice-based approaches is that 2D slices of a brain image lose the spatial information between each other since each 2D slice is processed independently. Sarraf et al. (2017), Wang S. H. et al. (2018), and Jain et al. (2019) adopted 2D MRI slices as the input type in their proposed model. Sarraf et al. (2017) used LeNet-5 as the CNN backbone and reported an accuracy of 96.86% for the classification of AD ad NC. Wang S. H. et al. (2018) trained their own 2D CNN from scratch. Jain et al. (2019) used 2D MRI slices as the input type in the model they presented. They adopted the VGG-16 pre-trained on ImageNet as the feature extractor. Lin et al. (2018) investigated to use CNN with PCA and Lasso to predict MCI-to-AD conversion. They trained the CNN as the feature extractor to input 2.5D patches, adopted PCA and Lasso to reduce the dimensions, and selected the most informative features. At last, fed the features to an extreme learning machine to make the classification. Furthermore, they tested the features generated from FreeSurfer together with the CNN-based features, and it turned out that using both features can generate better performance than using solely CNN-based or FreeSurfer-based features.

##### 3.4.2.2. 3D patch

3D patch-based approaches are like 2D slide-based methods, but instead of sampling the projections of particular planes cutting, the 3D brain scan into a set of 3D patches with stride as a hyperparameter. The sample size is larger after cutting. The 3D patch-based methods compensate for the loss of spatial information compared with 2D slice-based methods, but patches are often used independently during training. 3D patch-based methods need low memory when a model uses the same network for each patch. If training an independent network for each patch separately and then using an assemble architecture to integrate the results from previous independent networks, the complexity of the whole network will be high. Challenges in the 3D patch-based method are to choose the informative patches from the brain scan and select the most discriminative features. Qiu et al. (2020) and Zhang et al. (2021) adopted 3D patches as the input type.

##### 3.4.2.3. 3D ROI

3D ROI-based methods pay attention to specific regions which have been proved to be related to AD clinically. Images of ROI represent the 3D image of a segmented brain region. The selected regions, for example, gray matter volume, hippocampal volume, cortical thickness, etc., are usually informative. Using an ROI-based method will not lead to overfitting easily. The model interpretability is excellent since a human can see the contribution of each region in the model. The shortage of ROI-based methods is the prerequisite knowledge of the regions to select in AD. Liu et al. (2014) took the 3D ROI-based input and extracted features in Stacked sparse AEs. Li et al. (2015) adopted 3D ROI-based input in their model and used an SVM classifier.

##### 3.4.2.4. 3D subject

3D subject-based methods take a 3D brain scan as a whole, so complete integration of spatial information is preserved. Since a patient only provides one sample at a time, the sample size is too few compared with the number of subjects in popular datasets. Consequently, the risk of overfitting is high when using 3D subject-based methods. MRI scans are globally similar. Minor changes are not easily recognized in MRIs.

### 3.5. Autoencoder

An autoencoder (AE) is an artificial neural network in which the input and learning objectives are almost the same. Autoencoders aim to learn hidden representations of the input in an unsupervised manner. An autoencoder consists of an encoder and a decoder. Given input space and feature space, an autoencoder solves the mapping between the input and output to ensure the reconstruction error of the input feature is minimized. In other words, the latent layer feature, the encoded feature generated by the encoder, can be regarded as a representation of the input data.

The representational ability of an AE is limited. Stacked AEs are a combination of a series of AEs stacked together. In Stacked AEs, the output of hidden units of an AE is used as the input of another AE in the deeper layer. As the stacked AEs become deeper, the representational power increases. Stacked AEs can also be used in transfer learning. Stacked AEs as self-supervised learning can effectively extract the latent representation of input data. So stacked AEs can be used as a feature extractor. Train the AE with the training set, then replace the decoder with a classifier for classification purposes. The latent representation extracted in the AE can be used in pre-training. In tasks lacking datasets like AD classification and prediction, stacked AEs are pervasively used. Suk and Shen (2013), Suk et al. (2015), and Suk et al. (2016a) proposed networks used stacked AEs as feature extractors. SVM is used as the classifier to process the features to make the classification. Hosseini-Asl et al. (2016a,b) used a 3D CNN pre-trained with stacked 3D convolutional AEs in their work. Payan and Montana (2015) adopted sparse AEs and CNN and compared the classification accuracy of 2D and 3D approaches. The 3D approach provided a boost in performance compared to the 2D method.

### 3.6. Transformer

The utilization of state-of-the-art models of other computer vision tasks significantly improves the performance of AD classification and prediction. Integrating the latest model into AD-related studies is always a good idea. The next possible candidate to improve AD performance may be the attention mechanism. The attention mechanism proposed by Vaswani et al. (2017) was initially designed to solve Natural Language Processing (NLP) problems. Although the nature of the transformer is nothing but a weighted sum, the performance of the transformer is unbelievable and fabulous in a wide range of areas.

The vision transformer (Vit) proposed by Dosovitskiy et al. (2020) ditches the CNN structure and utilizes a pure transformer. As a new type of feature extractor, Vit focuses on patch-level attention instead of focusing on pixel-level attention. Vit achieves better performance than CNN in the various task in computer vision. If Vit is successfully used in AD diagnosis, the interpretability of the model will be increased since Vit depicts the importance of each area. The shortage of Vit is the dimension of the input feature is too large as most AD-related tasks use 3D images. Using Vit to handle such input with such a large dimension is unrealistic. Since 3D images contain much more spatial redundancy than 2D images and texts, it is necessary to reduce the duplication before processing.

With the great success of masked language models like Bidirectional Encoder Representations from Transformers (BERT) (Devlin et al., 2019) for pre-training in NLP, a new transfer learning method may also help improve performance. Masked Autoencoder (MAE), proposed by He et al. (2021), explains the natural difference between language and vision. Language is concrete and has high sematic information density, while vision is a continuous signal that contains duplication in space. Masked parts are more likely to be recovered in a vision task. An original image can be reconstructed based on the given partial observation information.

### 3.7. Transfer learning

Humans can utilize existing knowledge of one area to accelerate solving problems in another area. In many studies, researchers train their deep learning models from scratch. However, it is often inefficient since the training process is time-consuming, and a dataset of adequate size up to millions of images is required. Because of the high cost of learning directly from scratch, researchers expect to use existing knowledge to assist in learning new knowledge faster and better. Transfer learning means transferring knowledge learned from one domain to another. The source domain is defined as the domain that contains existing knowledge, while the target domain is the one to which the current knowledge is transferred. Since the most pervasively used backbone networks like LeNet, AlexNet, VGGNet, ResNet, DenseNet, and GoogLeNet are all trained on ImageNet, ImageNet has become the most common source dataset for transfer learning (Ardalan and Subbian, 2022). Researchers use transfer learning to pre-train their deep learning algorithms to solve the problem of scarcity of data samples.

Fine-tuning means applying a pre-trained model and using the weights of the pre-trained model to initialize the new model that will train. Fine-tuning helps to save a lot of time for training since a model does not need to train from scratch. Researchers can choose to freeze, fine-tune, and randomly initialize parts of the pre-train model. According to Ardalan and Subbian (2022), most researchers prefer to fine-tune convolution and fully connected layers.

The prediction for MCI conversion is more challenging than the classification between AD and HC because the brain structural changes of MCI may be very subtle. However, since the classification task between AD and HC is highly correlated with the task of MCI prediction, researchers often transfer the weights learned from the AD classification to initialize the parameters of the network for MCI classification. Khan et al. (2019) attempted to solve the need of large dataset issue with transfer learning. Their transfer learning strategy they deployed was to fine tune with layer-wise tuning which meant only a predefined group of layers were trained while other layers stayed frozen. Liu et al. (2021) adopted the AlexNet and GooLeNet as the base for transfer learning with an accuracy of 91.4 and 93.02%, respectively. The GoogLeNet achieved a slightly higher performance since it contains deeper layers and more convolutions than AlexNet. Odusami et al. (2021) utilized a transfer learning method for Alzheimer's detection. They utilized a pre-trained ResNet18 network as the source domain and unfroze all the layers to update the parameters of the network. Basaia et al. (2019) implemented transfer learning in the way that the weights of the CNN used to classify ADNI AD vs. HC were transferred to the other CNNs and used as pre-trained initial weights. Lian et al. (2020) transferred the weights learned from the AD vs. HC classification task to the MCI classification task. Hosseini-Asl et al. (2016a) pre-trained a 3D convolutional autoencoder in the source domain (CAD-Dementia) and fine-tuned in the target domain (ADNI). Li et al. (2015) pre-trained with RBM in an unsupervised manner. Similarly, Payan and Montana (2015) pre-trained convolutional layers with a sparse autoencoder and used the layers to initialize CNN.

## 4. Challenges and discussion

This article still contains some limitations. The papers we reviewed are mostly papers with high citations per year, which is not fair for newly published ones. The document and source types are strictly limited to “article” and “journal.” Furthermore, we only reviewed articles written in English. We mainly reviewed papers on Alzheimer's disease diagnosis using MRI as the data type. Neuroimaging of other forms, genetic, biological, voice-based, text-based, etc., may be reviewed in separate papers. The multi-modality models that can fuse information from different modalities usually outperform the models with only one modality since various modalities may contain complementary information.

Artificial intelligence, especially conventional machine learning and deep learning methods, is thriving in AD-related tasks. However, there are still some challenges. The datasets in the AD area are still small compared with datasets in computer vision tasks because of the privacy of medical data. Given the complexity of AD-related tasks, a large-scale dataset is a must for a researcher to develop more effective and powerful models. Currently, researchers mainly focus more on AD, MCI, and NC classification than prediction. The early detection of AD remains a challenging issue. Performance between each proposed model is hard to compare due to using different numbers of samples, modalities, pre-processing techniques, feature extractors, classifiers, etc.

### 4.1. Class imbalance

Class imbalance is a common issue in datasets. Usually, images in some classes may be far more than those in others in a dataset. Increasing the number of images that is fewer than other classes or reducing the number of images that is more than other classes are two methods to solve the imbalanced data issue. Synthetic Minority Oversampling Technique (SMOTE) technique is used to address the class imbalance problem in the dataset is by randomly duplicating the minority class of images in the dataset to minimize the overfitting problem (Chawla et al., 2002). Murugan et al. (2021) adopted SMOTE to overcome the class imbalance issue in their work and reported a training and validation accuracy of 99 and 94% compared with 96 and 78% when not implementing SMOTE. Data augmentation is one way to handle imbalanced data by enlarging the number of samples in the rare class. Reducing the number of images from the over-sampled class makes the dataset smaller. Afzal et al. (2019) adopted data augmentation to address the class imbalance concern in AD detection using 3D MRI images from OASIS and achieved high performance for Alzheimer's disease diagnosis. However, using a balanced dataset can improve the performance even if the dataset becomes smaller due to dataset balancing (Farooq et al., 2017). A balanced dataset is preferable. Another way of solving imbalanced class issues is by reconstructing medical images. Hu et al. (2020) proposed a Generative Adversarial Network (GAN) to reconstruct neuroimages. They used the new reconstructed images to augment the imbalanced dataset. They trained two 3D densely convolutional connected networks with the raw dataset and the fresh balanced and tested the performance of these two networks. The neuroimages generated from the GAN helped improve classification accuracy from 67 to 74%.

### 4.2. Data leakage

Data leakage refers to the use of testing data during training (Wen et al., 2020). Four main reasons that lead to data leakage are: incorrect data split, late split, improper transfer learning, and no independent test set. The late split occurs using data augmentation techniques before splitting the dataset into training, test, and validation sets. As a result, the images generated from the same source can be split into different datasets, leading to a biased evaluation. Incorrect data split means images of a subject at multiple time points are split into different training, test, and validation sets. Incorrect data split may occur when using 2D slices and 3D patches as deep learning input. The proper split should happen at the subject level. Prejudice transfer learning happens if the source and destination domain of transfer learning overlap. Different source and destination datasets are excellent ways to avoid prejudice transfer learning. No independent validation set exists in research in which the dataset is split into only training and test set. The test set should only be used for evaluation and never use the test set for hyperparameter optimization. A separate validation set that does not overlap with the test set can be used to optimize the hyperparameter of the model.

### 4.3. Trade-off discussion

In the reviewed articles of this paper, most authors utilized pre-processing techniques. Even though deep learning requires less pre-processing of data, for instance, Islam and Zhang (2018) and Khan et al. (2019) utilized no pre-processing techniques in their CNN networks; we still suggest pre-processing according to the standard pipeline before using the raw data, especially when adopting the conventional machine learning method. A recommend standard pre-processing pipeline includes: intensity correction, skull-stripping, registration, normalization, and tissue segmentation.

In the reviewed papers, SVM is the most pervasively utilized. However, the trend in recent years is that CNN will surge in popularity. Deep learning approaches achieved better performance in diagnostic tasks than conventional methods. A significant drawback of deep learning is it lacks interpretability and transparency. The deep learning models are in a black box state, and the internal operating mechanism is challenging to comprehend. Moreover, compared to conventional machine learning, deep learning techniques usually requires higher-performance graphics processing units, an enormous amount of storage, and more time to train.

Most of the research is conducted using one dataset. However, some researchers use more than one dataset for specific purposes. For instance, Liu et al. (2018) and Poloni and Ferrari (2022) used multiple datasets to enlarge the number of subjects. A few researchers use multiply datasets for different stages. Qiu et al. (2020) proposed a network to take ADNI as the training dataset and AIBL, FHS, and NACC as the testing dataset. Basaia et al. (2019) tested CNN on two datasets, ADNI and ADNI + Milan, and achieved an accuracy of 99% on ADNI and 98% ADNI + Milan in the classification of AD and HC, and an accuracy of 75% in detection cMCI and sMCI on both datasets. Lian et al. (2020) automated the identification of discriminative local patches and regions, then fused the features learned for classification by a hierarchical fully convolutional network on ADNI-1 and ADNI-2 and achieved an accuracy of 90.3% for AD vs. NC and 89.9% for pMCI vs. sMCI in the classification tasks.

Cutting the 3D image from various perspectives can generate 2D slices. 2D slice-based is a cheap method since the 2D image is much easier to process than 3D. In addition, slicing helps enlarge the sample size of the dataset. Usually, the 2D slices in the center with larger entropy will be selected, so the input dimension is further reduced. However, when using the slices of one 3D image independently, the interrelationship information may be lost through slicing. We recommend that researchers who do not have hardware support concentrate on designing a small architecture to try 2D slice-based data as the input form. Like the 2D slice-based methods, 3D patch-based methods provide a large dataset. The 3D patch compromises the 2D slice and the 3D subject image. However, the network should have to train a classifier for a patch. As a result, there will be too many classifiers to train. Extracting discriminative features and selecting the most informative ones from all the 3D patches is tough. Although the ROIs are usually informative, only one or a few regions will be considered in a model. However, AD often covers multiple brain regions. For researchers who comprehend how to define and use Region-of-Interests 3D ROI-based method may be a suitable solution with adequate interpretability. Subject-level methods contain only one sample per patient, so subject-level methods usually contain too few samples for a complicated task like AD detection.

There is no fixed answer to determining a suitable backbone or an input form. In general, larger and more complex models have a greater chance of yielding higher performance. According to Elharrouss et al. (2022), the complexity of DenseNet-121 and ResNet-101 is 0.525 a and 7.6 Giga Floating Point Operations Per Second (GFLOPs). The complexity of AlexNet is over ten times higher than ResNet-101. However, their top-1 error rates are 25.02% and 19.87%, which means fourteen times the complexity in exchange for a 5.15% reduction in the top-1 error rate.

Compared with CNN, one of the most significant advantages of the autoencoders is that it is an unsupervised learning method, and CNN must utilize marked data to work. However, autoencoders learn to capture as much information as possible, but the captured information may not be relevant to the specific task. If the information most pertinent to an issue makes up only a tiny part of the input, the autoencoders may lose much of it. Vision transformers outperform CNNs in some image classification tasks. However, Vision transformers need costly pre-training on large datasets. Researchers must choose the most suitable model based on their hardware conditions and specific application requirements, balancing performance and complexity.

## Author contributions

ZZ conceived the original idea for the review, performed the selection of paper and data extraction, prepared the table and figure, wrote the first draft of the manuscript, and contributed to the subsequent reviews and final version. KL, SD, and WB supervised the review process, revised the manuscript, and contributed to writing the final version. JC and C-OC helped conceptualize and supervise this study. MG, NW, and XW contributed to the literature review, result evaluation, and presentation. All authors participated in editing the first draft of the manuscript and read and approved the final manuscript.
